# On a Molecular Basis, Investigate Association of Molecular Structure with Bioactive Compounds, Anti-Nutritional Factors and Chemical and Nutrient Profiles of Canola Seeds and Co-Products from Canola Processing: Comparison Crusher Plants within Canada and within China as well as between Canada and China

**DOI:** 10.3390/nu10040519

**Published:** 2018-04-21

**Authors:** Walaa M. S. Gomaa, Gamal M. Mosaad, Peiqiang Yu

**Affiliations:** 1 Ministry of Strategic Research Chair Program, Department of Animal and Poultry Science, College of Agriculture and Bioresources, University of Saskatchewan, 51 Campus Drive, Saskatoon, SK S7N 5A8, Canada; walaasayed@aun.edu.eg; 2 Department of Animal Nutrition and Clinical Nutrition, Faculty of Veterinary Medicine, Assiut University, Assiut 71515, Egypt; mosaadgamal56@yahoo.com

**Keywords:** bioactive compounds, anti-nutritional factors, protein molecular structure, oil seeds, co-products from bio-oil processing, canola, crusher plants, Canada and China

## Abstract

The objectives of this study were to: (1) Use molecular spectroscopy as a novel technique to quantify protein molecular structures in relation to its chemical profiles and bioenergy values in oil-seeds and co-products from bio-oil processing. (2) Determine and compare: (a) protein molecular structure using Fourier transform infrared (FT/IR-ATR) molecular spectroscopy technique; (b) bioactive compounds, anti-nutritional factors, and chemical composition; and (c) bioenergy values in oil seeds (canola seeds), co-products (meal or pellets) from bio-oil processing plants in Canada in comparison with China. (3) Determine the relationship between protein molecular structural features and nutrient profiles in oil-seeds and co-products from bio-oil processing. Our results showed the possibility to characterize protein molecular structure using FT/IR molecular spectroscopy. Processing induced changes between oil seeds and co-products were found in the chemical, bioenergy profiles and protein molecular structure. However, no strong correlation was found between the chemical and nutrient profiles of oil seeds (canola seeds) and their protein molecular structure. On the other hand, co-products were strongly correlated with protein molecular structure in the chemical profile and bioenergy values. Generally, comparisons of oil seeds (canola seeds) and co-products (meal or pellets) in Canada, in China, and between Canada and China indicated the presence of variations among different crusher plants and bio-oil processing products.

## 1. Introduction

In the international economy, canola has become a central issue as the second most abundant oil source [1] with the valuable co-product of oil extraction: high quality protein rich meal [2]. Canola was modified from rapeseed [3] by Canadian plant breeders [4] to obtain plant with low levels of “erucic acid” in the oil (<2% of total fatty acids in the oil) and low levels of glucosinolates in the non-oil part (<30 μmol in its defatted meal) [5,6,7]. Therefore, rapeseeds that contain low levels of erucic acid in oil and glucosinolates in meal are called canola in North America and “double-zero” rapeseeds in Europe [6,8,9]. About 13% of the total oilseed and protein meals production in the world comes from canola seeds and rapeseeds [10] and the biggest producers of them in the world are China and Canada [11].

Although canola has been extensively studied [4,12], the relation between its protein molecular structure and the chemical and nutrient profiles remains unclear. In addition, little information is present about canola variations among the different crusher plants, the bio-oil processing products, and the different producers’ countries as Canada and China. These variations affect the molecular structure, chemical composition, and concentration of protein and carbohydrates in canola seeds and meal [13,14] and availability of nutrients in the meal [5].

Thus, the main purposes of the present study were to use molecular spectroscopy as a novel technique to quantify protein molecular structure of canola in relation to its chemical and energy profiles and to compare the protein molecular structure, chemical profile and energy values in canola seeds, meal and pellets from different crushing plants in the main two producer countries: Canada and China.

The hypothesis of this study was that the protein molecular structure changes induced by processing had close relationship to the chemical and bioenergy profiles in canola seeds, meal and pellets and the chemical and bioenergy profiles could be predicted using the parameters of protein molecular structure.

## 2. Materials and Methods

### 2.1. Sample Preparation

Systematic sampling process was arranged by Canada Council of Canola (CCC, Winnipeg, Manitoba, Canada). Oil seeds (canola seeds) and co-products from bio-oil processing (canola meal or meal pellets) were obtained from five different bio-oil processing plants in Canada as well as from five different bio-oil processing plants in China (three different batches of seeds, meal or meal pellet produced at different times were obtained from each plant). Total samples: Canadian seed: 5 × 3 = 15; Canadian meal: 3 × 2 = 6; Canadian meal pellet: 3 × 3 = 9; Chinese seed: 5 × 3 = 15; and Chinese meal: 5 × 3 = 15.

### 2.2. Chemical Analysis

Canola seeds were ground by a coffee grinder (PC770, Loblaws Inc., Toronto, ON, Canada) for 20 s while canola meal and pellets were ground via a 1 mm screen using Retsch ZM 200 rotor mill (Rose Scientific Ltd., Edmonton, AB, Canada) and analyzed for dry matter (DM) (AOAC official method 930.15), ash (AOAC official method 942.05), crude protein (CP) (AOAC official method 984.13), crude fat (EE) (AOAC official method 920.39), neutral detergent fiber (NDF) (AOAC official method 2002.04), acid detergent fiber (ADF) (AOAC official method 973.18) and acid detergent lignin (ADL) (AOAC official method 973.18) according to AOAC [15]. Neutral detergent-insoluble crude protein (NDICP), acid detergent-insoluble crude protein (ADICP) and non-protein nitrogen (NPN) were analyzed according to Licitra et al. [16]. Soluble crude protein (SCP) was analyzed in accordance with Roe et al. [17]. Structural and non-structural carbohydrates were determined using Van Soest et al. [18] and NRC [19]. Total carbohydrate (CHO), non-fiber CHO (NFC), hemicellulose, and cellulose were calculated as follows: CHO = 100 − EE − CP − ash; NFC = 100 − (NDF − NDICP) − EE − CP − ash; hemicellulose = NDF − ADF; and cellulose = ADF − ADL according to NRC [19]. All samples were analyzed in duplicate and repeated if error exceeded 5%.

### 2.3. BioEnergy Values

The available bioenergy is very important in ration formulation [5] and it can be estimated by using total digestible nutrient (TDN), as well as digestible energy, metabolizable energy, and net energy [20]. The truly digestible non-fiber carbohydrate (td NFC), total digestible crude protein (td CP), total digestible neutral detergent fiber (td NDF), and total digestible fatty acid (td FA) were calculated according to NRC [19] based on canola chemical composition.

Total digestible nutrient at maintenance (TDN_1x_), digestible energy at a production level (DE_3x_), metabolizable energy at a production level (ME_3x_), and net energy at a production level (NE_3x_) were estimated using NRC [19]. Metabolizable energy, net energy for maintenance (NE_m_), net energy for gain (NE_g_) were predicted using NRC [21].

### 2.4. Protein Molecular Structure

Attenuated Total Reflectance (ATR)—Fourier transform infrared (FT/IR) molecular spectroscopy can be used as a rapid tool to detect protein molecular structures [22,23]. The protein molecular spectrum data of canola seeds, meal and pellets were collected using ATR-FT/IR vibrational spectroscopy 4200 (JASCO Corporation, Tokyo, Japan), at the Feed/Food Molecular Structure Analysis Lab, University of Saskatchewan (Saskatchewan, SK, Canada). The samples were ground through a 1 mm screen using a coffee grinder (PC770, Loblaws Inc., Toronto, ON, Canada) before spectral analysis. The IR spectrum of each sample was obtained within the mid-IR range (ca. 4000–800 cm^−1^) with 32 scans at a resolution of 4 cm^−1^. Five replicates were randomly carried out for each sample. The spectral data were analyzed by OMNIC 7.3 software (Spectra Tech., Madison, WI, USA). Amide I (ca. 1650) and amide II (ca. 1550) were detected. For protein 2nd structure of α-helix (ca. 1657) and β-sheet (ca. 1630) in the IR regions of approximately 1715 to 1480 cm^−1^, two steps were applied as described by Yu [24]. The ratios of amide I and II and α-helix and β-sheet spectral intensities were calculated. The ratio was obtained by the height or area under one functional group band (e.g., amide I) divided by the height or area under another functional group band (amide II) at each pixel, which represents the biological component ratio intensity and distribution in the tissue.

### 2.5. Statistical Analysis

Effect of plant crusher on chemical and nutrient profile data of canola seeds, meal and pellets in both Canada and China were analyzed using the mixed model procedure of SAS 9.1.4 (SAS Institute, Cary, NC, USA).The model used for the analysis is as follows:

Y_ij_ = μ + P_i_ + e_ij_ (1)

where Y_ij_ is an observation of the dependent variable ij, μ is the population mean for the variable, and P_i_ is the effect of plant crushers within Canada (i = 1, 2, 3, 4, 5, total five crushers) or within China (i = A, B, C, D, E, total five crushers). Three batches from three different processing times in each crusher were replicates. e_ij_ is the random error associated with observation ij.

The residual analysis was carried out to check the model assumptions. Normality check was carried out using Proc Univariate with Normal and Plot options in SAS. For all statistical analyses, significance was declared at *p* < 0.05. Treatment means were compared using Tukey method. Contrast was carried out between meal and pellets.

Correlation Analysis between the protein molecular structure and chemical profile and bioenergy values was analyzed using the CORR procedure of SAS (Version 9.4, SAS Institute, Cary, NC, USA). The normality checking for correlation analysis was done using Univariate Procedure with Plot and Normal option. Multiple regression analysis of protein molecular structure spectral profile with chemical and nutrient profile were performed using PROC REG procedure of SAS 9.4 (SAS Institute, Cary, NC, USA). The model variables selection for regression was carried out using Stepwise Option.

## 3. Results and Discussion

### 3.1. Effect of Different Bio-Oil Processing Plants on Chemical Analysis within Canada and China: Comparison between Canada and China

The chemical composition of canola seeds: comparisons of crusher plants within Canada and within China as well as between Canada and China are presented in Table 1. Canola seeds in Canada had significantly higher dry matter, non-protein nitrogen, neutral detergent-insoluble crude protein, acid detergent fiber, and cellulose and significantly lower contents of soluble crude protein, sugar, and non-structural carbohydrates than canola seeds in China. There were significant differences in dry matter, ash, soluble crude protein, non-protein nitrogen, neutral detergent-insoluble crude protein, acid detergent-insoluble crude protein, sugar, and acid detergent lignin among the five different crusher plants within Canada. The Chinese crusher plants showed significant differences only in dry matter, crude protein, and acid detergent-insoluble crude protein. The variations in chemical profiles among crusher plants and between canola in Canada and China may be related to inherent variations in the type of seeds.

Total carbohydrate, neutral detergent fiber, acid detergent fiber, non-fiber carbohydrates, non-structural carbohydrates and ether extract of canola seeds were not significantly affected by the different crushing plants within Canada or China. Results for dry matter, ash, and CHO were similar to those reported by Samadi et al. [25] while the values of crude protein, soluble crude protein, neutral detergent-insoluble crude protein, acid detergent-insoluble crude protein, neutral detergent fiber, and acid detergent fiber were different than their results.

Table 2 shows the chemical composition of canola meals: canola meal in Canada had significantly higher dry matter, ash, neutral detergent-insoluble crude protein, neutral detergent fiber, acid detergent lignin, hemicellulose and non-fiber carbohydrates and significantly lower crude protein, soluble crude protein, non-protein nitrogen, sugar, and cellulose compared with those in China. We found that ash, crude protein, CHO, and cellulose contents of canola meal in our study are consistent with the finding of Xin and Yu [20]. Brito and Broderick [26] reported lower values for neutral detergent fiber (23.7%), acid detergent fiber (15.8%), and hemicellulose (7.87%) than our study. Maison [10] analyzed canola meal and his results for dry matter, crude protein and ash agree with our findings.

Moreover, the same table indicated that, ash, soluble crude protein, non-protein nitrogen, neutral detergent-insoluble crude protein, acid detergent-insoluble crude protein, sugar, neutral detergent fiber, hemicellulose, cellulose, non-fiber carbohydrates and non-structural carbohydrates had significant differences among crusher plants within Canada and China. Based on contrast P value between meal and pellets in Canada, we found significant differences in ash, soluble crude protein, non-protein nitrogen (%SCP), neutral detergent-insoluble crude protein, acid detergent-insoluble crude protein, neutral detergent fiber, acid detergent fiber, acid detergent lignin, hemicellulose and cellulose contents.

Our results in Table 1 and Table 2 reflect that the processing induced variations in the chemical profile between seeds and meal and among bio-oil processing products in the different crushing plants within Canada and within China and between Canada and China.

### 3.2. Effect of Different Bio-Oil Processing Plants on BioEnergy Values within Canada and China: Comparison between Canada and China

As shown in Table 3, no significant differences were detected in digestible nutrients or energy values between canola seeds in Canada and China. The same for crushing plants within Canada and China which did not show any significant differences in digestible nutrients or energy values except for td CP, which was significantly different among the different crusher plants within China. Our results were parallel with the published data of Samadi et al. [25].

Results for the energy values of canola meal are presented in Table 4. Canola meal in China had significantly higher td NDF, td CP, TDN _1x_, TDN p _3x_, TDN p _4x_, DE _1x_, DE p _3x_, ME p _3x_, NEL p _3x_, ME _3x_, NE m _3x_, and NE g _3x_. This may indicate that Chinese canola might be better than Canadian canola as the energy source. Our results are in the range of those reported by Theodoridou and Yu [27] and Xin and Yu [20]. Regarding crusher plants within Canada, a significant difference was found in all digestible nutrients and all energy values, while no significant difference was detected among crusher plants within China. Based on contrast P-values, td NDF, TDN _1x_, TDN p _3x_, TDN p _4x_, DE _1x_, DE p _3x_, ME p _3x_, NEL p _3x_, ME _3x_, NE m_3x_, and NE g _3x_ showed significant differences between meal and pellets. Bell [5] indicated that the variation in energy values in canola meal may be related to several factors as variety and quality of seeds, methods of processing, and the content of fiber in addition to the environmental factors. There were differences between the energy values of seeds and meal that might indicate the effect of oil extraction during canola meal processing. After pressing and solvent extraction during canola meal processing, it has less than 1% oil (CCC, Canada), which affects the energy values of meal.

Moreover, Toghyani et al. [28] indicated that Variations in oil, protein and fiber contents of canola may affect its energy content.

### 3.3. Effect of Different Crusher Plants on Protein Molecular Structure within Canada and China: Comparison between Canada and China

Variations between seeds and bio-oil processing product (meal) in the protein molecular structure indicated processing induced changes during canola meal manufacture. The cause of these changes in protein structure between meal and seeds may be the denaturation or disorganization that occurs to protein molecules during processing [29]. Table 5 shows the protein molecular structure characteristics of canola seeds detected by FT/IR molecular spectroscopy. Canola seeds in Canada and China, and crusher plants within Canada aand within China showed no significant differences in protein molecular structure except the ratio of α-helix/β-sheet which showed significant differences among crusher plants within Canada. Samadi and Yu [30] reported that changes in α-helix/β-sheet ratio can occur as a result of denaturation of α-helix and β-sheet from heat treatment during processing.

Amide I band is very sensitive to the protein secondary structure [20]. In our result, there were no variations in α-helix and β-sheet among the crusher plants within Canada and china, as no variations was found in amide I among the different crusher plants. Zhang and Yu [31] in their synchrotron-based study recorded these values for canola seeds: amide I: 16.77; amide II: 5.64; area ratios of amide I and II: 3.01; α-helix height: 0.25; β-sheet height: 0.22; and ratio α-helix/β-sheet: 1.15.

Protein molecular structure characteristics of canola meal are shown in Table 6. We found lower IR absorbance (*p* < 0.05) in amide I and II peak area, amide I peak area, area ratios of amide I and II, amide I height, height ratios of amide I and II, α-helix height, β-sheet height, and ratio α-helix to β-sheet for canola meal in Canada than canola meal in China. It might be suggested from these results that canola meal in Canada and China differ in their protein utilization related to the differences in their protein molecular structure. Yu [29] indicated that heat treatment during feed processing changes the protein secondary structure and these changes could affect the protein utilization and availability. Moreover, Xin and Yu [20] indicated presence of a close relationship between protein secondary structure (α-helix and β-sheet) and protein quality, availability and digestibility and protein utilization may be decreased due to increasing the percentage of β-sheet. No differences were found among crusher plants within Canada in the protein molecular structure except in area ratios of amide I and II. Crusher plants within China showed significant differences in amide I and II peak area, amide I peak area, amide II peak area, area ratios of amide I and II, amide I height, height ratios of amide I and II, alpha-helix height, beta-sheet height, and ratio a helix: b sheet. Amide I peak area, amide I height, α-helix height, and β-sheet height showed significant differences between meal and pellets. Area ratio amide I: amide II and ratio α-helix: β-sheet of canola meal are in the range of those reported by Theodoridou and Yu [4]. However, Xin and Yu [20] reported lower values for amide I area and height, amide II area and height, a helix and b sheet than our study and this might be due to the different sources, batches, storage condition or time of processing of canola in the two experiment.

Xin and Yu [20] said that “the amide I and II bands are the two primary features within the protein spectrum. The amide I band is particularly sensitive to changes in protein secondary structure, and α-helix and β-sheet are the two typical structures in protein secondary structure, which closely relates to nutritional quality, digestive behavior, and nutrient availability. In protein secondary structure, a higher percentage of β-sheet may cause lower protein degradability and utilization”.

### 3.4. Relationship Study between Protein Structure Spectral Characteristics and Chemical and Nutrient Profiles

#### 3.4.1. Correlation Study between Protein Structure Spectral Characteristics and Chemical Profile

We performed correlation study to indicate that the nutritional values of canola seeds and meal may be related partially to the protein molecular structure characteristics. The correlation analysis between protein structure spectral characteristics and chemical profile of canola seeds (Table 7) indicated that no strong correlation was found between them.

Table 8 shows the correlation analysis between protein structure characteristics and chemical profile of canola meal. Regarding protein profile, CP had a positive correlation with α-helix height (*r* = 0.61, *p* < 0.001) while, SCP was positively correlated with amide I area (*r* = 0.67, *p* < 0.001), amide I height (*r* = 0.73, *p* < 0.001), α-helix height (*r* = 0.83, *p* < 0.001), and β-sheet height (*r* = 0.60, *p* < 0.001). NPN showed strong positive correlation with amide I area (*r* = 0.71, *p* < 0.001), amide I height (*r* = 0.75, *p* < 0.001), and α-helix height (*r* = 0.78, *p* < 0.001). However, only α-helix height (*r* = −0.67, *p* < 0.001) showed a negative correlation with NDICP. In carbohydrate profile, ADF (%NDF) was positively correlated with amide I height (*r* = 0.68, *p* < 0.001) and β-sheet height (*r* = 0.75, *p* < 0.001), while hemicellulose showed a negative correlation with them (*r* = −0.66, *p* < 0.001 and *r* = −0.74, *p* < 0.001, respectively).

#### 3.4.2. Correlation Study between Protein Structure Spectral Characteristics and Energy Profile

We did not observe strong correlation between the protein structure characteristics and energy profile in canola seeds (Table 9) or canola meal (Table 10) except td CP of canola meal showed a positive correlation with α-helix height (*r* = −0.61, *p* < 0.001).

### 3.5. Regression Study between Protein Structure Spectral Characteristics and Chemical and Nutrient Profile in Canola Seed or Canola Meal

#### 3.5.1. Regression Study between Protein Structure Spectral Characteristics and Chemical Profile

Multiple regression analyses were conducted to select variables to predict nutrient profiles. The tested multiple regression model was Y = Amide I and II peak area (AI_II_T) + amide I area (AI) + amide I height (AIH) + amide II area (AII) + amide II height (AIIH) + area ratio of amide I to amide II (R_AAI_II) + height ratio of amide I to amide II (R_H_AI_II) + α-helix height (Alpha) + β-sheet height (Beta) + height ratio of α-helix to β-sheet (R_Ha_b), with variables (*p* < 0.05) selected to leave in the prediction equation. Table 11 shows regression analyses for predicting chemical profile of canola seeds. Height ratio of amide I to amide II was left in the model as a predictor for DM and ADL, while amide I and II peak area and amide I area could be used as predictors for SCP, NPN (%SCP) and NDICP.

For predicting chemical profile of canola meal (Table 12), α-helix height could be used to predict DM and NDICP. Ash and NFC (%CHO) were predicted by amide II height and height ratio of α-helix to β-sheet while, the height ratio of α-helix to β-sheet was a single predictor for CP and sugar (%NFC). In addition, height ratio of amide I to amide II was left in the model as a single predictor for CHO and ADL (%DM). Amide II height, amide II area and β-sheet height could be left in the model as a single predictor for ADF (%DM), ADL (%NDF), ADF (%NDF), and Cellulose respectively. Height ratio of amide I to amide II and α-helix height were left in the model to detect SCP. Height ratio of amide I to amide II, α-helix height and amide I and II peak area could be used to detect NPN (%SCP). In summary, protein structure spectral features could be used as predictors for the chemical profile of canola meal.

#### 3.5.2. Regression Study between Protein Structure Spectral Characteristics and Energy Profile

We found that no variables met the 0.05 significance level for entry into the model to detect energy values of canola seeds (Table 13). Multiple regression analyses for predicting energy values of canola meal are shown in Table 14. Height ratio of amide I to amide II could be used as a predictor for digestible energy at one times maintenance, digestible energy at a productive level of intake, metabolizable energy at production level of intake, net energy for lactation at productive level, metabolizable energy, net energy for maintenance, and net energy for gain. Truly digestible neutral detergent fiber and truly digestible crude protein could be predicted by height ratio of α-helix to β-sheet. However, amide II area and height ratio of α-helix to β-sheet could be variables to predict truly digestible non-fiber carbohydrate. Total digestible nutrients were predicted by the area ratio of amide I to amide II.

## 4. Conclusions

Based on the results mentioned above, it was indicated that protein molecular structure of canola could be characterized on a molecular basis using FT/IR molecular spectroscopy and chemical and nutrient profiles could be correlated to its protein molecular structure. In addition, the chemical profile, and inherent molecular structures of canola meal were affected by the bio-processing during its manufacture. Canola seeds in Canada had different chemical profile and protein molecular structure, when compared with canola seeds in China but we did not detect a difference between them in the bioenergy profile. On the other hand, canola meal in Canada and China were different in chemical profile, energy values, and protein molecular structures. Concerning comparison crusher plants within Canada, within China, and between meal and pellets within Canada, our result reflected variations among bio-oil processing products among the different crushing plants within Canada and within China. Generally, crusher plants within Canada showed variations in the chemical profile (seeds and meal) and bioenergy values (meal). The seeds from crusher plants within China showed variations in dry matter, crude protein, and acid detergent-insoluble crude protein from chemical profile, td CP from bioenergy values, and in protein molecular structures. However, the meal from the same crusher plants within China were different in the chemical profile, tdNDF, tdNFC, tdCP from energy profile, and protein molecular structures. Regarding the comparison of meal versus pellets in Canada, values were significantly different in the chemical profile, bioenergy profile, and protein molecular structures (amide I, amide I height, α-helix height, and β-sheet height). We only found a strong correlation between canola meal and protein molecular structures in some parameters in the chemical profile, and bioenergy profile. Further study should be done to study the relationship between their protein molecular structure and protein utilization and availability.

## Figures and Tables

**Table 1:** Bioactive compounds, anti-nutritional factors, and chemical composition of oil-seeds from bio-oil processing (canola seeds): Comparison of crusher plants within Canada and within China as well as between Canada and China.

Items	Crusher Plants within Canada							Crusher Plants within China							Overall			
	C1	C2	C3	C4	C5	* SEM	*p*-Value	A	B	C	D	E	SEM	*p*-Value	Canada	China	SEM	*p*-Value
Basic chemical																		
** DM (%)	93.08 ^b^	94.70 ^a^	92.94 ^b^	91.91 ^c^	91.66 ^c^	0.181	<0.001	92.14 ^a^	91.96 ^a^	92.12 ^a^	92.22 ^a^	91.45 ^b^	0.087	<0.001	92.86 ^a^	91.98 ^b^	0.217	0.008
Ash (%DM)	3.98 ^a^	3.72 ^b^	4.00 ^a^	3.83 ^a,b^	3.73 ^b^	0.052	0.007	3.73	3.82	3.82	3.84	3.86	0.042	0.293	3.85	3.81	0.030	0.393
EE (%DM)	43.19	42.58	45.32	45.22	43.58	1.315	0.510	42.33	46.27	45.32	43.65	44.47	0.969	0.115	43.98	44.41	0.547	0.582
FA (%DM)	42.19	41.58	44.32	44.22	42.58	1.315	0.510	41.33	45.27	44.32	42.65	43.47	0.969	0.115	42.98	43.41	0.547	0.582
Protein profile																		
CP (%DM)	23.17	21.95	22.83	21.92	22.02	0.325	0.062	22.35 ^a^	21.48 ^b^	22.14 ^a,b^	22.16 ^a,b^	22.02 ^a,b^	0.166	0.035	22.38	22.03	0.149	0.110
SCP (%DM)	11.27 ^a,b,c^	9.67 ^c^	10.81 ^b,c^	12.22 ^a,b^	12.81 ^a^	0.351	<0.001	13.51	12.72	13.46	11.79	12.98	0.857	0.628	11.36 ^b^	12.89 ^a^	0.344	0.004
SCP (%CP)	48.64 ^b^	44.06 ^b^	47.38 ^b^	55.71 ^a^	58.20 ^a^	1.261	<0.001	60.50	59.20	60.78	53.08	58.96	3.760	0.613	50.80 ^b^	58.50 ^a^	1.552	0.002
NPN (%DM)	8.68	8.30	8.24	8.10	6.62	0.820	0.482	5.34	5.32	2.70	3.18	2.95	0.985	0.206	7.99 ^a^	3.90 ^b^	0.430	<0.001
NPN (%CP)	37.44	37.84	36.13	36.89	30.10	3.641	0.572	23.88	24.87	12.17	14.29	13.40	4.576	0.207	35.68 ^a^	17.72 ^b^	1.951	<0.001
NPN (%SCP)	76.96 ^a,b^	85.87 ^a^	76.12 ^a,b^	66.15 ^a,b^	51.86 ^b^	6.075	0.024	40.15	43.33	20.19	26.55	22.91	8.743	0.294	71.39 ^a^	30.63 ^b^	4.002	<0.001
NDICP (%DM)	2.61 ^a^	2.65 ^a^	2.29 ^b^	2.27 ^b^	1.98 ^c^	0.060	<0.001	2.04	2.07	2.01	2.02	1.98	0.111	0.981	2.36 ^a^	2.03 ^b^	0.058	<0.001
NDICP (%CP)	11.26 ^a,b^	12.09 ^a^	10.04 ^b,c^	10.37 ^b,c^	9.00 ^c^	0.305	0.001	9.14	9.67	9.10	9.14	9.01	0.523	0.904	10.55 ^a^	9.21 ^b^	0.261	0.001
ADICP (%DM)	1.20 ^a^	1.10 ^a,b^	0.95 ^b^	1.18 ^a^	1.14 ^a^	0.034	0.003	1.04 ^b^	1.19 ^a^	1.17 ^a,b^	1.22 ^a^	1.17 ^a,b^	0.032	0.017	1.11	1.16	0.024	0.207
ADICP (%CP)	5.18 ^a^	5.04 ^a^	4.16 ^b^	5.41 ^a^	5.16 ^a^	0.184	0.006	4.64 ^b^	5.54 ^a^	5.29 ^a,b^	5.52 ^a^	5.34 ^a,b^	0.159	0.015	4.99	5.27	0.121	0.121
Carbohydrate profile																		
CHO (%DM)	29.66	31.76	27.85	29.04	30.67	1.436	0.413	31.50	28.52	28.72	30.34	29.66	0.958	0.240	29.79	29.75	0.566	0.956
Sugar (%DM)	4.72	4.53	5.40	5.97	5.89	0.476	0.185	7.67	5.76	7.30	5.99	5.42	1.020	0.474	5.30 ^b^	6.43 ^a^	0.362	0.037
Sugar (%NFC)	30.96 ^a,b^	28.10 ^b^	38.73 ^a,b^	44.05 ^a^	36.44 ^a,b^	3.061	0.029	42.62	42.65	52.26	39.78	37.37	5.979	0.502	35.66 ^b^	42.94 ^a^	2.297	0.033
NDF (%DM)	16.94	18.07	16.18	17.68	16.46	0.533	0.137	15.93	16.71	16.67	17.34	17.04	0.712	0.703	17.06	16.74	0.288	0.428
ADF (%DM)	12.43	12.77	12.16	13.20	12.16	0.371	0.295	11.76	12.26	11.38	11.85	12.09	0.372	0.542	12.54 ^a^	11.87 ^b^	0.169	0.009
ADF (%NDF)	73.34	70.90	75.21	74.70	73.89	2.001	0.607	74.09	73.32	68.27	68.57	71.02	1.810	0.148	73.61	71.05	0.896	0.053
ADL (%DM)	5.48 ^b^	5.82 ^a,b^	4.99 ^b^	6.58 ^a^	5.80 ^a,b^	0.193	0.002	5.29	6.11	6.36	5.97	5.78	0.258	0.116				
ADL (%NDF)	32.37 ^a,b^	32.31 ^a,b^	30.82 ^b^	37.22 ^a^	35.24 ^a,b^	1.282	0.032	33.24	36.72	38.14	34.59	33.92	1.630	0.254	33.61	35.32	0.787	0.136
Hemicellulose (%DM)	4.51	5.30	4.01	4.48	4.31	0.455	0.403	4.17	4.45	5.29	5.49	4.95	0.478	0.317	4.52	4.87	0.216	0.263
Cellulose (%DM)	6.95	6.95	7.18	6.62	6.35	0.272	0.296	6.46	6.15	5.03	5.87	6.31	0.438	0.230	6.81 ^a^	5.96 ^b^	0.177	0.002
NFC (%DM)	15.33	16.34	13.97	13.63	16.18	1.208	0.423	17.62	13.89	14.06	15.03	14.60	0.838	0.060	15.09	15.04	0.514	0.943
NFC (%CHO)	51.69	50.97	49.89	46.98	52.79	2.003	0.360	55.84	48.58	48.91	49.62	49.19	1.764	0.073	50.46	50.43	0.957	0.982
NSC (%DM)	4.72	4.53	5.40	5.97	5.89	0.476	0.185	7.67	5.76	7.30	5.99	5.42	1.020	0.474	5.30 ^b^	6.43 ^a^	0.362	0.037

Notes: * SEM, standard error of the mean. ** DM, dry matter; EE, ether extract; FA, fatty acid; CP, crude protein; SCP, soluble crude protein; NPN, non-protein nitrogen; NDICP, neutral detergent-insoluble crude protein; ADICP, acid detergent-insoluble crude protein; CHO, total carbohydrate; NDF, neutral detergent fiber; ADF, acid detergent fiber; ADL, acid detergent lignin; Hemicellulose = NDF − ADF; Cellulose =ADF − ADL; NFC, non-fiber carbohydrate; NSC, non-structural carbohydrate. Means in the same row with different letters (a,b,c) differ significantly (*p* < 0.05).

**Table 2:** Bioactive compounds, anti-nutritional factors and chemical composition of co-products from bio-oil processing (canola meal): Comparison of crusher plants within Canada and within China as well as between Canada and China.

Items	Crusher Plants within Canada							Contrast *p* Value	Crusher Plants within China							Overall (Meal Only)			
	C1 Meal	C2 Meal	C3 Pellet	C4 Pellet	C5 Pellet	* SEM	*p* Value	Meal vs. Pellet	A	B	C	D	E	SEM	*p* Value	Canada	China	SEM	*p* Value
Basic chemical																			
** DM (%)	89.69	89.03	88.72	89.84	88.99	0.432	0.352	0.664	88.23	88.52	88.50	88.16	88.78	0.273	0.532	89.36 ^a^	88.44 ^b^	0.196	0.004
Ash (%DM)	7.67 ^b,c^	8.29 ^a^	8.19 ^a,b^	7.46 ^c^	7.27 ^c^	0.124	0.0006	0.013	7.09 ^b^	7.12 ^b^	7.38 ^a^	7.14 ^a,b^	7.33 ^a,b^	0.053	0.009	7.98 ^a^	7.21 ^b^	0.086	0.001
Protein profile																			
CP (%DM)	42.42 ^a^	40.86 ^b^	41.62 ^a,b^	41.78 ^a,b^	41.77 ^a,b^	0.277	0.033	0.752	42.41	43.31	43.04	43.42	41.91	0.331	0.041	41.64 ^b^	42.82 ^a^	0.284	0.009
SCP (%DM)	7.33 ^b^	7.40 ^b^	8.56 ^a,b^	9.34 ^a^	7.72 ^a,b^	0.385	0.018	0.008	10.05 ^b^	9.81 ^b,c^	11.48 ^a^	10.12 ^a,b^	8.46 ^c^	0.305	0.001	7.37 ^b^	9.99 ^a^	0.356	<0.001
SCP (%CP)	17.28 ^b^	18.08 ^b^	20.57 ^a,b^	22.34 ^a^	18.49 ^a,b^	0.838	0.010	0.005	23.73 ^a,b^	22.66 ^b^	26.67 ^a^	23.32 ^a,b^	20.19 ^b^	0.773	0.002	17.68 ^b^	23.31 ^a^	0.793	<0.001
NPN (%DM)	7.13 ^a,b^	6.90 ^a,b^	7.89 ^a,b^	8.33 ^a^	6.04 ^b^	0.437	0.031	0.335	8.45 ^b^	8.93 ^b^	10.06 ^a^	8.69 ^b^	7.53 ^b^	0.276	0.001	7.02 ^b^	8.63 ^a^	0.33	0.003
NPN (%CP)	16.79 ^a,b^	16.86 ^a,b^	18.96 ^a,b^	19.93 ^a^	14.45 ^b^	0.973	0.020	0.308	19.95 ^b^	19.38 ^b^	23.38 ^a^	20.01 ^b^	17.97 ^b^	0.718	0.004	16.83 ^b^	20.14 ^a^	0.741	0.005
NPN (%SCP)	96.90 ^a^	93.07 ^a^	92.21 ^a^	89.23 ^a^	78.15 ^b^	1.870	0.001	0.001	84.00	85.56	87.69	85.86	89.01	1.233	0.109	94.98 ^a^	86.43 ^b^	1.089	<0.001
NDICP (%DM)	7.78 ^a,b^	9.03 ^a^	5.93 ^b^	5.95 ^b^	7.99 ^a,b^	0.558	0.010	0.006	4.13 ^c^	6.59 ^a^	4.08 ^c^	5.30 ^b^	5.83 ^b^	0.135	<0.001	8.40 ^a^	5.19 ^b^	0.381	<0.001
NDICP (%CP)	18.32 ^a,b^	22.14 ^a^	14.25 ^b^	14.24 ^b^	19.12 ^a,b^	1.405	0.011	0.007	9.74 ^c^	15.21 ^a^	9.49 ^c^	12.20 ^b^	13.90 ^a^	0.288	<0.001	20.23 ^a^	12.11 ^b^	0.941	<0.001
ADICP (%DM)	2.35 ^a^	2.27 ^a,b^	1.90 ^c^	2.23 ^b^	2.19 ^b^	0.024	<0.001	<0.001	2.08 ^b^	2.76 ^a^	2.08 ^b^	2.06 ^b^	2.30 ^a,b^	0.100	0.002	2.31	2.25	0.092	0.659
ADICP (%CP)	5.54 ^a^	5.56 ^a^	4.56 ^b^	5.34 ^a^	5.22 ^a^	0.082	<0.001	<0.001	4.90 ^b^	6.37 ^a^	4.83 ^b^	4.75 ^b^	5.48 ^a,b^	0.232	0.003	5.55	5.27	0.212	0.357
Carbohydrate profile																			
CHO (%DM)	49.90 ^a^	50.84 ^a^	50.19 ^a^	50.76 ^a^	50.96 ^a,b,c^	0.248	0.052	0.275	50.49	49.57	49.58	49.44	50.76	0.330	0.053	50.37	49.97	0.245	0.261
Sugar (%DM)	7.62 ^a,b^	8.35 ^a,b^	8.61 ^a^	7.20 ^b^	8.30 ^a,b^	0.263	0.018	0.832	8.33 ^b^	7.99 ^b^	8.22 ^b^	8.44 ^b^	10.62 ^a^	0.322	0.001	7.98	8.72	0.335	0.137
Sugar (%NFC)	28.75 ^b,c^	32.24 ^a,b^	31.01 ^a,b,c^	27.93 ^c^	33.03 ^a^	0.919	0.012	0.853	32.41 ^b^	31.89 ^b^	34.26 ^b^	35.53 ^a,b^	41.07 ^a^	1.346	0.005	30.50 ^b^	35.03 ^a^	1.224	0.017
NDF (%DM)	31.18 ^b^	33.97 ^a^	28.35 ^c^	30.96 ^b^	33.82 ^a^	0.541	<0.001	0.011	28.90 ^b^	31.11 ^a^	29.66 ^a,b^	31.20 ^a^	30.71 ^a^	0.323	0.003	32.58 ^a^	30.28 ^b^	0.431	0.001
ADF (%DM)	19.94 ^bc^	21.84 ^a^	19.32 ^c^	22.06 ^a^	20.94 ^a,b^	0.259	<0.001	0.622	20.98	21.45	20.46	20.61	19.93	0.370	0.124	20.89	20.69	0.299	0.637
ADF (%NDF)	63.94 ^b^	64.37 ^b^	68.17 ^a,b^	71.34 ^a^	61.92 ^b^	1.488	0.008	0.052	72.59 ^a^	68.96 ^a,b^	69.00 ^a,b^	66.49 ^b,c^	64.88 ^c^	0.802	0.001	64.16 ^b^	68.39 ^a^	0.935	0.005
ADL (%DM)	9.41 ^b^	10.45 ^a^	7.78 ^c^	10.22 ^a^	9.20 ^b^	0.131	<0.001	0.0001	9.08 ^a,b^	10.01 ^a^	8.44 ^b^	8.85 ^b^	8.49 ^b^	0.239	0.006	9.93 ^a^	8.97 ^b^	0.228	0.008
ADL (%NDF)	30.19 ^b^	30.80 ^b^	27.47 ^c^	33.04 ^a^	27.21 ^c^	0.472	<0.001	0.016	31.4 ^a,b^	32.18 ^a^	28.46 ^b,c^	28.55 ^b,c^	27.66 ^c^	0.762	0.006	30.49	29.65	0.652	0.373
Hemicellulose (%DM)	11.24 ^a,b^	12.12 ^a^	9.04 ^b^	8.90 ^b^	12.88 ^a^	0.607	0.002	0.029	7.91^c^	9.66 ^a,b^	9.20 ^b^	10.39 ^a^	10.78 ^a^	0.244	0.001	11.68 ^a^	9.59 ^b^	0.369	0.001
Cellulose (%DM)	10.53^b^	11.39 ^a,b^	11.53 ^a,b^	11.84 ^a^	11.73 ^a^	0.231	0.017	0.006	11.90	11.44	12.02	11.76	11.44	0.303	0.571	10.96 ^b^	11.71 ^a^	0.176	0.007
NFC (%DM)	26.49 ^a,b^	25.90 ^b,c^	27.77 ^a^	25.75 ^b,c^	25.12 ^c^	0.291	0.001	0.946	25.72 ^a^	25.05 ^a,b^	24.01 ^b^	23.74 ^b^	25.87 ^a^	0.310	0.002	26.20 ^a^	24.88 ^b^	0.31	0.008
NFC (%CHO)	53.10 ^a,b^	50.94 ^b,c^	55.33 ^a^	50.72 ^b,c^	49.30 ^c^	0.521	<0.001	0.626	50.94 ^a^	50.55 ^a,b^	48.43 ^a,b^	48.02 ^b^	50.97 ^a^	0.553	0.007	52.02 ^a^	49.78 ^b^	0.528	0.007
NSC (%DM)	8.62 ^a,b^	9.35 ^a,b^	9.61 ^a^	8.20 ^b^	9.30 ^a,b^	0.263	0.018	0.832	9.33 ^b^	8.99 ^b^	9.22 ^b^	9.44 ^b^	11.62 ^a^	0.322	0.001	8.98	9.72	0.335	0.137

Notes: * SEM, standard error of the mean. ** DM, dry matter; CP, crude protein; SCP, soluble crude protein; NPN, non-protein nitrogen; NDICP, neutral detergent-insoluble crude protein; ADICP, acid detergent-insoluble crude protein; CHO, total carbohydrate; NDF, neutral detergent fiber; ADF, acid detergent fiber; ADL, acid detergent lignin; Hemicellulose = NDF − ADF; Cellulose = ADF − ADL; NFC, non-fiber carbohydrate; NSC, non-structural carbohydrate. Means in the same row with different letters (a,b,c) differ significantly (*p* < 0.05).

**Table 3:** Digestible nutrients and bioenergy values of canola seeds: Comparison of crusher plants within Canada and within China as well as between Canada and China.

Items	Crusher Plants within Canada							Crusher Plants within China							Overall			
	C1	C2	C3	C4	C5	* SEM	*p* Value	A	B	C	D	E	SEM	*p* Value	Canada	China	SEM	*p* Value
Digestible nutrients % of DM																		
** td NDF	3.14	3.45	3.30	2.87	2.99	0.246	0.500	3.06	2.84	2.66	3.29	3.29	0.282	0.464	3.15	3.03	0.117	0.464
td NFC	15.02	16.02	13.69	13.35	15.86	1.184	0.422	17.27	13.62	13.78	14.73	14.31	0.821	0.060	14.79	14.74	0.504	0.947
td CP	22.69	21.51	22.45	21.44	21.57	0.328	0.060	21.93 ^a^	21.00 ^b^	21.67 ^a,b^	21.67 ^a,b^	21.55 ^a,b^	0.170	0.032	21.93	21.57	0.152	0.099
td FA	42.19	41.58	44.32	44.22	42.58	1.315	0.510	41.33	45.27	44.32	42.65	43.47	0.969	0.115	42.98	43.41	0.547	0.582
TDN _1X_	128.79	127.52	132.17	130.16	129.23	1.832	0.501	128.25	132.31	130.82	128.66	129.94	1.456	0.340	129.57	130.00	0.745	0.689
TDN p _3X_	118.28	117.11	121.38	119.54	118.68	1.683	0.502	117.78	121.52	120.15	118.16	119.34	1.336	0.338	119.00	119.39	0.684	0.688
TDN p _4X_	113.02	111.90	115.98	114.22	113.40	1.608	0.502	112.55	116.11	114.81	112.91	114.03	1.278	0.341	113.71	114.08	0.654	0.687
Energy values (Mcal/kg DM)																		
DE _1x_	5.70	5.63	5.84	5.74	5.70	0.079	0.490	5.67	5.82	5.77	5.68	5.73	0.062	0.418	5.72	5.74	0.032	0.779
DE p _3x_	5.23	5.17	5.36	5.27	5.24	0.072	0.464	5.20	5.35	5.30	5.22	5.26	0.056	0.395	5.26	5.27	0.029	0.821
ME p _3x_	5.02	4.95	5.16	5.07	5.03	0.079	0.500	4.99	5.15	5.10	5.00	5.06	0.061	0.377	5.05	5.06	0.032	0.745
NEL p _3x_	3.62	3.57	3.74	3.67	3.63	0.068	0.509	3.59	3.74	3.69	3.61	3.66	0.052	0.338	3.64	3.66	0.027	0.744
ME _3x_	4.67	4.61	4.79	4.71	4.67	0.065	0.474	4.65	4.77	4.73	4.66	4.70	0.050	0.402	4.69	4.70	0.026	0.760
NE m_3x_	3.34	3.30	3.43	3.37	3.34	0.049	0.502	3.32	3.42	3.38	3.33	3.36	0.038	0.346	3.36	3.36	0.020	0.831
NE g _3x_	2.43	2.40	2.50	2.45	2.43	0.038	0.496	2.42	2.49	2.47	2.42	2.45	0.029	0.416	2.44	2.45	0.015	0.735
DE p _4x_	5.00	4.94	5.12	5.04	5.00	0.068	0.472	4.97	5.11	5.06	4.99	5.03	0.053	0.413	5.02	5.03	0.027	0.784
ME p _4x_	4.79	4.72	4.92	4.83	4.79	0.076	0.486	4.75	4.91	4.86	4.77	4.82	0.059	0.402	4.81	4.82	0.030	0.782
NEL p _4x_	3.45	3.40	3.56	3.49	3.45	0.065	0.514	3.41	3..56	3.51	3.44	3.48	0.050	0.309	3.47	3.48	0.026	0.761

Notes: * SEM, standard error of the mean. ** td NDF, truly digestible neutral detergent fiber; td NFC, truly digestible non-fiber carbohydrate; td CP, truly digestible crude protein; td FA, truly digestible fatty acid; TDN _1x_, total digestible nutrients at one times maintenance; TDN p _3x_, total digestible nutrients at productive level of intake at three times maintenance; TDN p _4x_, total digestible nutrients at productive level at four times maintenance; DE _1x_, digestible energy at one times maintenance; DE p _3x_, digestible energy at a productive level of intake (3x maintenance); ME p _3x_, metabolizable energy at production level of intake (3x maintenance); NEL p _3x_, net energy for lactation at productive level (3x maintenance); ME _3x_, metabolizable energy; NE m _3x_, net energy for maintenance; NE g _3x_, net energy for gain. Means in the same row with different letters (a,b) differ significantly (*p* < 0.05).

**Table 4:** Digestible nutrients and bioenergy values of co-products from bio-oil processing (canola meal): Comparison crusher plants within Canada and within China as well as between Canada and China.

Items	Crusher Plants within Canada							Contrast *p* Value	Crusher Plants within China							Overall (Meal Only)			
	C1 Meal	C2 Meal	C3 Pellet	C4 Pellet	C5 Pellet	* SEM	*p* Value	Meal vs. Pellet	A	B	C	D	E	SEM	*p* Value	Canada	China	SEM	*p* Value
Digestible nutrients % of DM																			
** td NDF	4.78 ^c^	4.787 ^c^	5.560 ^b^	4.987 ^b,c^	6.203 ^a^	0.132	<0.001	<0.001	5.75 ^a,b^	4.91 ^b^	6.72 ^a^	6.43 ^a^	6.29 ^a^	0.232	0.002	4.78 ^b^	6.02 ^a^	0.221	0.001
td NFC	25.97 ^a,b^	25.38 ^b,c^	27.22 ^a^	25.23 ^b,c^	24.62 ^c^	0.285	<0.001	0.962	25.21 ^a^	24.55 ^a,b^	23.53 ^b^	23.27 ^b^	25.35 ^a^	0.304	0.002	25.68 ^a^	24.38 ^b^	0.304	0.007
td CP	41.49 ^a^	39.95 ^b^	40.86 ^a,b^	40.88 ^a,b^	40.90 ^a,b^	0.282	0.040	0.540	41.58 ^a,b^	42.20 ^a,b^	42.21 ^a,b^	42.59 ^a^	40.99 ^b^	0.328	0.042	40.72 ^b^	41.92 ^a^	0.281	0.007
TDN _1X_	65.23 ^b^	63.12 ^d^	66.64 ^a^	64.11 ^c,d^	64.73 ^b,c^	0.239	<0.001	0.001	65.55	64.66	65.46	65.29	65.64	0.288	0.198	64.18 ^b^	65.32 ^a^	0.270	0.007
TDN p _3X_	59.90 ^b^	57.97 ^d^	61.20 ^a^	58.87 ^c,d^	59.45 ^b,c^	0.219	<0.001	0.001	60.20	59.38	60.12	59.96	60.28	0.263	0.191	58.94 ^b^	59.99 ^a^	0.248	0.008
TDN p _4X_	57.24 ^b^	55.40 ^d^	58.48 ^a^	56.25 ^c,d^	56.81 ^b,c^	0.209	<0.001	0.001	57.52	56.74	57.44	57.30	57.60	0.253	0.198	56.32 ^b^	57.32 ^a^	0.237	0.008
Energy values (Mcal/kg DM)																			
DE _1x_	3.31 ^a,b^	3.21 ^c^	3.36 ^a^	3.26 ^b,c^	3.29 ^b^	0.012	<0.001	0.003	3.33	3.30	3.34	3.33	3.32	0.016	0.539	3.26 ^b^	3.33 ^a^	0.013	0.003
DE p _3x_	3.04 ^a,b^	2.94 ^c^	3.09 ^a^	3.00 ^b^	3.02 ^b^	0.011	<0.001	0.002	3.06	3.03	3.06	3.06	3.05	0.016	0.563	2.99 ^b^	3.05 ^a^	0.013	0.004
ME p _3x_	2.62 ^a,b^	2.52 ^c^	2.67 ^a^	2.58 ^b^	2.60 ^b^	0.001	<0.001	0.002	2.64	2.61	2.64	2.64	2.63	0.016	0.563	2.57 ^b^	2.63 ^a^	0.013	0.004
NEL p _3x_	1.65 ^a,b^	1.58 ^c^	1.69 ^a^	1.62 ^b^	1.64 ^b^	0.008	<0.001	0.001	1.66	1.65	1.67	1.67	1.66	0.010	0.546	1.62 ^b^	1.66 ^a^	0.009	0.002
ME _3x_	2.72 ^a,b^	2.63 ^c^	2.76 ^a^	2.67 ^b^	2.69 ^b^	0.010	<0.001	0.002	2.73	2.71	2.74	2.73	2.73	0.014	0.573	2.67 ^b^	2.73 ^a^	0.011	0.003
NE m_3x_	1.80 ^a,b^	1.72 ^c^	1.83 ^a^	1.76 ^b,c^	1.77 ^b^	0.010	0.001	0.009	1.81	1.79	1.81	1.81	1.80	0.011	0.541	1.76 ^b^	1.80 ^a^	0.010	0.004
NE g _3x_	1.17 ^a,b^	1.10 ^d^	1.20 ^a^	1.13 ^c^	1.15 ^b,c^	0.007	<0.001	0.003	1.18	1.16	1.18	1.18	1.18	0.010	0.627	1.14 ^b^	1.18 ^a^	0.009	0.005
DE p _4x_	2.91 ^a,b^	2.81 ^d^	2.95 ^a^	2.86 ^c,d^	2.88 ^b,c^	0.011	<0.001	0.003	2.92	2.90	2.93	2.92	2.92	0.014	0.577	2.86 ^b^	2.92 ^a^	0.012	0.003
ME p _4x_	2.49 ^a,b^	2.39 ^c^	2.53 ^a^	2.44 ^b,c^	2.46 ^b^	0.011	<0.001	0.003	2.50	2.48	2.51	2.50	2.50	0.014	0.573	2.44 ^b^	2.50 ^a^	0.012	0.003
NEL p _4x_	1.56 ^a,b^	1.49 ^d^	1.59 ^a^	1.52 ^c,d^	1.54 ^b,c^	0.007	<0.001	0.003	1.57	1.55	1.57	1.57	1.57	0.010	0.627	1.53 ^b^	1.57 ^a^	0.009	0.005

Notes: * SEM, standard error of the mean. ** td NDF, truly digestible neutral detergent fiber; td NFC, truly digestible non-fiber carbohydrate; td CP, truly digestible crude protein; td FA, truly digestible fatty acid; TDN _1x_, total digestible nutrients at one times maintenance; TDN p _3x_, total digestible nutrients at productive level of intake at three times maintenance; TDN p _4x_, total digestible nutrients at productive level at four times maintenance; DE _1x_, digestible energy at one times maintenance; DE p _3x_, digestible energy at a productive level of intake (3x maintenance); ME p _3x_, metabolizable energy at production level of intake (3x maintenance); NEL p _3x_, net energy for lactation at productive level (3x maintenance); ME _3x_, metabolizable energy; NE m _3x_, net energy for maintenance; NE g _3x_, net energy for gain. Means in the same row with different letters (a,b,c,d) differ significantly (*p* < 0.05).

**Table 5:** Protein molecular structure characteristics of oil-seeds from bio-oil processing (canola seeds): Comparison of crusher plants within Canada and within China as well as between Canada and China.

Items	Crusher Plants within Canada							Crusher Plants within China							Overall			
	C1	C2	C3	C4	C5	* SEM	*p* Value	A	B	C	D	E	SEM	*p* Value	Canada	China	SEM	*p* Value
Amide I and II peak area	44.16	41.44	43.19	45.90	48.02	2.197	0.328	42.76	45.10	48.13	44.15	54.01	3.941	0.339	44.54	46.83	1.486	0.286
Amide I	21.24	20.20	20.48	22.54	22.23	1.002	0.422	19.80	20.43	22.17	20.59	25.27	1.904	0.322	21.34	21.65	0.707	0.757
Amide II	6.26	6.44	5.79	6.36	6.26	0.464	0.869	5.66	5.16	6.32	6.43	6.85	0.571	0.307	6.22	6.08	0.231	0.679
Area ratios of amide I and II	3.48	3.16	3.66	3.92	3.72	0.297	0.484	3.70	4.09	3.81	3.24	3.82	0.398	0.667	3.59	3.73	0.151	0.508
Amide I Height	0.324	0.310	0.311	0.343	0.329	0.015	0.562	0.290	0.297	0.321	0.310	0.354	0.021	0.296	0.32	0.31	0.008	0.454
Amide II Height	0.138	0.142	0.132	0.136	0.129	0.009	0.845	0.120	0.111	0.131	0.133	0.134	0.010	0.442	0.14	0.13	0.004	0.099
Height ratios of amide I and II	2.39	2.20	2.40	2.61	2.62	0.107	0.095	2.49	2.73	2.50	2.38	2.71	0.165	0.527	2.45	2.56	0.066	0.223
α-helix height	0.304	0.291	0.277	0.310	0.304	0.015	0.575	0.263	0.267	0.298	0.281	0.336	0.023	0.236	0.30	0.29	0.009	0.526
β-sheet height	0.280	0.261	0.268	0.306	0.286	0.013	0.202	0.244	0.252	0.272	0.264	0.304	0.017	0.214	0.28	0.27	0.008	0.247
Ratio α-helix: β-sheet	1.083 ^a,b^	1.121 ^a^	1.037 ^b^	1.013 ^b^	1.064 ^a,b^	0.018	0.014	1.077	1.058	1.093	1.063	1.102	0.025	0.693	1.06	1.08	0.011	0.355

Notes: * SEM, standard error of the mean. Means in the same row with different letters differ significantly (*p* < 0.05).

**Table 6:** Protein molecular structure characteristics of co-products from bio-oil processing (canola meal): Comparison of crusher plants within Canada and within China as well as between Canada and China.

Items	Crusher Plants within Canada							Contrast *p* Value	Crusher Plants within China							Overall (Meal Only)			
	C1 Meal	C2 Meal	C3 Pellet	C4 Pellet	C5 Pellet	* SEM	*p* Value	Meal vs. Pellet	A	B	C	D	E	SEM	*p* Value	Canada	China	SEM	*p* Value
Amide I and II peak area	52.98	54.06	57.22	58.91	54.25	2.118	0.312	0.121	56.10 ^a,b^	58.78 ^a^	57.67 ^a,b^	59.81 ^a^	53.85 ^b^	1.035	0.016	53.52 ^b^	57.24 ^a^	1.138	0.032
Amide I	23.35	24.29	25.98	26.74	24.31	0.852	0.097	0.038	25.49 ^a,b^	26.76 ^a,b^	26.54 ^a,b^	27.08 ^a^	24.30 ^b^	0.548	0.028	23.82 ^b^	26.04 ^a^	0.518	0.007
Amide II	7.52	7.59	7.75	8.34	8.02	0.327	0.425	0.138	8.06 ^a^	8.04 ^a^	7.94 ^a^	8.00 ^a^	7.21 ^b^	0.187	0.041	7.56	7.85	0.182	0.269
Area ratios of amide I and II	3.12 ^b^	3.20 ^a,b^	3.36 ^a^	3.21 ^a,b^	3.04 ^b^	0.045	0.006	0.369	3.17 ^b^	3.33 ^a,b^	3.35 ^a^	3.39 ^a^	3.38 ^a^	0.038	0.015	3.16 ^b^	3.32 ^a^	0.033	0.003
Amide I Height	0.327	0.345	0.369	0.375	0.349	0.013	0.163	0.044	0.370 ^a,b^	0.376 ^a,b^	0.377 ^a,b^	0.381 ^a^	0.344 ^b^	0.007	0.032	0.336 ^b^	0.370 ^a^	0.008	0.006
Amide II Height	0.160	0.166	0.168	0.177	0.178	0.009	0.579	0.193	0.178	0.175	0.166	0.170	0.161	0.004	0.059	0.163	0.170	0.004	0.258
Height ratios of amide I and II	2.06	2.08	2.20	2.12	1.97	0.055	0.117	0.626	2.07 ^b^	2.15 ^a,b^	2.28 ^a^	2.25 ^a,b^	2.14 ^a,b^	0.042	0.036	2.07 ^b^	2.18 ^a^	0.033	0.028
α-helix height	0.289	0.300	0.328	0.334	0.315	0.012	0.103	0.016	0.332 ^b^	0.346 ^a,b^	0.350 ^a,b^	0.362 ^a^	0.321 ^b^	0.006	0.008	0.295 ^b^	0.342 ^a^	0.007	<0.001
β-sheet height	0.286	0.305	0.332	0.333	0.307	0.012	0.099	0.030	0.331 ^a^	0.332 ^a^	0.325 ^a,b^	0.328 ^a,b^	0.302 ^b^	0.006	0.027	0.295 ^b^	0.324 ^a^	0.007	0.008
Ratio α-helix: β-sheet	1.013	0.984	0.987	1.007	1.030	0.018	0.365	0.567	1.005 ^b^	1.043 ^a,b^	1.077 ^a^	1.105 ^a^	1.063 ^a,b^	0.014	0.005	0.999 ^b^	1.058 ^a^	0.013	0.005

Notes: * SEM, standard error of the mean. Means in the same row with different letters (a,b) differ significantly (*p* < 0.05).

**Table 7:** Correlation analyses between protein structure spectral characteristics and chemical profile of canola seeds.

Items	** AI_II_T		AI		AII		R_AAI_II		AIH		AIIH		R_HAI_II		Alpha		Beta		R_Ha_b	
	*** *r* Value	*p* Value	*R* Value	*p* Value	*r* Value	*p* Value	*R* Value	*p* Value	*r* Value	*p* Value	*R* Value	*p* Value	*r* Value	*p* Value	*r* Value	*p* Value	*r* Value	*p* Value	*r* Value	*p* Value
* DM (%)	−0.45	0.012	−0.33	0.072	−0.07	0.704	−0.28	0.133	−0.22	0.251	0.18	0.356	−0.54	0.002	−0.19	0.328	−0.29	0.116	0.22	0.235
Ash (%DM)	−0.10	0.614	−0.03	0.866	0.02	0.913	0.00	0.986	0.01	0.973	0.04	0.821	0.02	0.939	−0.01	0.965	0.09	0.621	−0.17	0.368
EE (%DM)	0.22	0.242	0.16	0.402	−0.13	0.496	0.29	0.122	0.09	0.624	−0.11	0.561	0.26	0.161	0.05	0.786	0.06	0.748	−0.11	0.578
FA (%DM)	0.22	0.242	0.16	0.402	−0.13	0.496	0.29	0.122	0.09	0.624	−0.11	0.561	0.26	0.161	0.05	0.786	0.06	0.748	−0.11	0.578
Protein profile																				
CP (%DM)	0.08	0.659	0.18	0.337	0.12	0.518	0.00	0.985	0.25	0.188	0.36	0.053	−0.16	0.402	0.23	0.219	0.29	0.120	−0.08	0.682
SCP (%DM)	0.52	0.003	0.39	0.033	0.20	0.282	0.09	0.642	0.27	0.151	0.04	0.840	0.23	0.225	0.26	0.160	0.20	0.279	0.09	0.656
SCP (%CP)	0.52	0.003	0.37	0.042	0.18	0.346	0.12	0.539	0.23	0.215	−0.03	0.890	0.28	0.130	0.24	0.211	0.18	0.347	0.07	0.716
NPN (%DM)	−0.30	0.106	−0.15	0.433	−0.21	0.277	0.07	0.722	0.00	0.996	0.08	0.684	−0.11	0.582	−0.05	0.803	0.03	0.867	−0.26	0.173
NPN (%CP)	−0.32	0.081	−0.18	0.347	−0.23	0.217	0.08	0.673	−0.03	0.860	0.02	0.914	−0.09	0.646	−0.09	0.657	−0.01	0.954	−0.24	0.202
NPN (%SCP)	−0.41	0.025	−0.25	0.181	−0.16	0.394	−0.06	0.740	−0.09	0.637	0.13	0.502	−0.28	0.140	−0.11	0.572	−0.07	0.723	−0.11	0.549
NDICP (%DM)	−0.52	0.003	−0.32	0.090	−0.16	0.400	−0.08	0.686	−0.15	0.425	0.07	0.722	−0.26	0.170	−0.16	0.413	−0.09	0.634	−0.12	0.528
NDICP (%CP)	−0.57	0.001	−0.39	0.036	−0.18	0.335	−0.11	0.579	−0.22	0.235	−0.01	0.980	−0.25	0.183	−0.22	0.253	−0.16	0.408	−0.12	0.538
ADICP (%DM)	−0.00	0.982	0.03	0.875	0.10	0.601	−0.11	0.547	0.07	0.709	−0.05	0.792	0.12	0.518	0.04	0.835	0.11	0.573	−0.10	0.608
ADICP (%CP)	0.00	1.000	0.01	0.971	0.07	0.705	−0.08	0.664	0.03	0.890	−0.13	0.489	0.19	0.326	−0.01	0.959	0.05	0.777	−0.09	0.644
CHO profile																				
CHO (%DM)	−0.19	0.304	−0.17	0.363	0.10	0.596	−0.24	0.201	−0.15	0.432	−0.03	0.866	−0.14	0.475	−0.12	0.535	−0.14	0.477	0.11	0.575
Sugar (%DM)	0.36	0.054	0.28	0.128	0.26	0.173	−0.06	0.751	0.19	0.316	0.15	0.444	−0.10	0.613	0.16	0.392	0.17	0.367	−0.03	0.869
Sugar (%NFC)	0.45	0.014	0.36	0.049	0.23	0.226	0.02	0.923	0.27	0.156	0.16	0.394	0.01	0.941	0.21	0.255	0.26	0.164	−0.17	0.365
NDF (%DM)	−0.34	0.068	−0.18	0.344	0.05	0.780	−0.16	0.393	−0.08	0.692	0.00	0.984	−0.11	0.572	−0.09	0.648	0.00	0.990	−0.05	0.796
ADF (%DM)	−0.30	0.113	−0.10	0.600	0.02	0.900	−0.08	0.683	0.05	0.814	0.08	0.672	−0.13	0.486	−0.01	0.977	0.16	0.408	−0.14	0.477
ADF (%NDF)	0.08	0.678	0.11	0.561	0.06	0.738	0.02	0.906	0.15	0.443	0.17	0.367	−0.12	0.525	0.12	0.538	0.21	0.276	−0.06	0.771
ADL (%DM)	0.12	0.514	0.13	0.484	−0.08	0.677	0.22	0.233	0.05	0.801	−0.24	0.205	0.37	0.047	0.03	0.871	0.08	0.692	−0.06	0.767
ADL (%NDF)	0.35	0.060	0.28	0.137	−0.07	0.711	0.31	0.093	0.13	0.510	−0.21	0.275	0.43	0.019	0.15	0.441	0.13	0.490	0.05	0.799
Hemi (%DM)	−0.14	0.468	−0.12	0.529	0.01	0.973	−0.10	0.593	−0.13	0.506	−0.12	0.534	0.06	0.737	−0.10	0.617	−0.15	0.424	0.04	0.816
Cell (%DM)	−0.33	0.074	−0.17	0.383	0.08	0.694	−0.23	0.215	−0.01	0.968	0.24	0.209	−0.38	0.037	−0.05	0.803	0.05	0.800	−0.03	0.858
NFC (%DM)	−0.18	0.347	−0.21	0.273	−0.01	0.952	−0.12	0.517	−0.20	0.301	−0.07	0.725	−0.13	0.492	−0.15	0.445	−0.22	0.233	0.26	0.162
NFC (%CHO)	−0.10	0.606	−0.15	0.426	−0.08	0.667	0.00	0.994	−0.17	0.372	−0.07	0.713	−0.12	0.533	−0.10	0.608	−0.22	0.239	0.33	0.078
NSC (%DM)	0.36	0.054	0.28	0.128	0.26	0.173	−0.06	0.751	0.19	0.316	0.15	0.444	−0.10	0.613	0.16	0.392	0.17	0.367	−0.03	0.869

Notes: * DM, dry matter; CP, crude protein; SCP, soluble crude protein; NPN, non-protein nitrogen; NDICP, neutral detergent-insoluble crude protein; ADICP, acid detergent-insoluble crude protein; CHO, total carbohydrate; NDF, neutral detergent fiber; ADF, acid detergent fiber; ADL, acid detergent lignin; Hemi, Hemicellulose (Hemicellulose = NDF − ADF); Cell, Cellulose (Cellulose = ADF − ADL); NFC, non-fiber carbohydrate; NSC, non-structural carbohydrate. ** AI_II_T, Amide I and II peak area; AI, Amide I area; AII, Amide II area; R_AAI_II, Area ratios of amide I and II; AIH, Amide I height; AIIH, Amide II height; R_HAI_II, Height ratios of amide I and II; alpha, α-helix height; beta, β-sheet height; R_Ha_b, Ratio α-helix:β-sheet. *** *r*: correlation coefficient calculated using Spearman method.

**Table 8:** Correlation analyses between protein structure spectral characteristics and chemical profile of canola meal.

Items	** AI_II_T		AI		AII		R_AAI_II		AIH		AIIH		R_HAI_II		Alpha		Beta		R_Ha_b	
	*** *r* Value	*p* Value	*R* Value	*p* Value	*r* Value	*p* Value	*R* Value	*p* Value	*r* Value	*p* Value	*r* Value	*p* Value	*r* value	*p* Value	*r* Value	*p* Value	*r* Value	*p* Value	*r* Value	*p* Value
* DM (%)	−0.27	0.155	−0.19	0.319	−0.07	0.707	−0.27	0.142	−0.34	0.066	−0.22	0.237	−0.07	0.718	−0.38	0.038	−0.33	0.079	−0.18	0.345
Ash (%DM)	−0.25	0.177	−0.26	0.163	−0.34	0.070	−0.05	0.797	−0.39	0.035	−0.33	0.075	0.01	0.954	−0.55	0.002	−0.29	0.123	−0.52	0.004
Protein profile																				
CP (%DM)	0.40	0.028	0.42	0.021	0.22	0.251	0.32	0.081	0.43	0.017	0.05	0.786	0.33	0.075	0.61	<0.001	0.29	0.119	0.58	0.001
SCP (%DM)	0.56	0.001	0.67	<0.0001	0.34	0.063	0.49	0.006	0.73	<0.0001	0.08	0.693	0.59	0.001	0.83	<0.0001	0.60	<0.001	0.46	0.010
SCP (%CP)	0.56	0.001	0.67	<0.0001	0.35	0.058	0.47	0.009	0.74	<0.0001	0.10	0.588	0.57	0.001	0.82	<0.0001	0.61	0.001	0.44	0.014
NPN (%DM)	0.63	0.000	0.71	<0.0001	0.39	0.033	0.45	0.014	0.75	<0.0001	0.10	0.586	0.59	0.001	0.78	<0.0001	0.63	<0.001	0.36	0.050
NPN (%CP)	0.59	0.001	0.68	<0.0001	0.34	0.066	0.45	0.012	0.70	<0.0001	0.05	0.809	0.62	0.000	0.73	<0.0001	0.59	0.001	0.33	0.078
NPN (%SCP)	−0.08	0.673	−0.14	0.449	−0.22	0.233	0.03	0.873	−0.22	0.243	−0.24	0.197	0.05	0.803	−0.39	0.034	−0.11	0.557	−0.40	0.029
NDICP (%DM)	−0.42	0.021	−0.45	0.012	−0.21	0.264	−0.42	0.021	−0.57	0.001	−0.09	0.635	−0.47	0.009	−0.67	<0.0001	−0.54	0.002	−0.37	0.046
NDICP (%CP)	−0.43	0.018	−0.47	0.009	−0.22	0.236	−0.43	0.019	−0.58	0.001	−0.08	0.656	−0.48	0.008	−0.68	<0.0001	−0.54	0.002	−0.38	0.038
ADICP (%DM)	−0.13	0.501	−0.13	0.486	0.08	0.691	−0.28	0.128	−0.19	0.311	0.11	0.549	−0.41	0.025	−0.32	0.087	−0.27	0.155	−0.18	0.345
ADICP (%CP)	−0.17	0.381	−0.16	0.397	0.04	0.851	−0.26	0.160	−0.21	0.272	0.09	0.618	−0.42	0.020	−0.34	0.063	−0.28	0.133	−0.22	0.242
CHO profile																				
CHO (%DM)	−0.41	0.026	−0.41	0.024	−0.07	0.700	−0.43	0.017	−0.38	0.039	0.12	0.519	−0.54	0.002	−0.50	0.005	−0.31	0.090	−0.37	0.045
Sugar (%DM)	−0.27	0.154	−0.25	0.185	−0.45	0.012	0.25	0.182	−0.15	0.418	−0.31	0.096	0.10	0.600	0.03	0.855	−0.20	0.282	0.28	0.132
Sugar (%NFC)	−0.14	0.457	−0.07	0.694	−0.28	0.129	0.36	0.049	−0.02	0.930	−0.33	0.072	0.22	0.243	0.29	0.122	−0.18	0.343	0.58	0.001
NDF (%DM)	−0.32	0.082	−0.33	0.078	−0.06	0.740	−0.39	0.031	−0.44	0.016	−0.07	0.713	−0.40	0.028	−0.40	0.027	−0.52	0.003	−0.05	0.781
ADF (%DM)	0.15	0.434	0.12	0.520	0.41	0.026	−0.38	0.039	0.16	0.397	0.41	0.026	−0.42	0.022	−0.04	0.829	0.16	0.395	−0.28	0.139
ADF (%NDF)	0.55	0.002	0.54	0.002	0.46	0.011	0.13	0.508	0.68	<0.001	0.45	0.013	0.15	0.419	0.51	0.004	0.75	<0.001	−0.08	0.689
ADL (%DM)	0.01	0.947	0.01	0.978	0.30	0.112	−0.49	0.006	−0.01	0.975	0.37	0.042	−0.51	0.004	−0.22	0.254	−0.06	0.745	−0.29	0.125
ADL (%NDF)	0.30	0.105	0.28	0.137	0.41	0.025	−0.25	0.181	0.33	0.074	0.49	0.006	−0.27	0.156	0.09	0.640	0.35	0.060	−0.25	0.187
Hemi (%DM)	−0.51	0.004	−0.52	0.003	−0.38	0.038	−0.20	0.278	−0.66	<0.001	−0.37	0.043	−0.25	0.184	−0.51	0.004	−0.74	<0.001	0.04	0.821
Cell (%DM)	0.30	0.104	0.24	0.211	0.31	0.092	0.03	0.888	0.33	0.071	0.20	0.290	0.08	0.667	0.29	0.126	0.38	0.040	−0.01	0.953
NFC (%DM)	−0.33	0.072	−0.39	0.032	−0.29	0.121	−0.23	0.221	−0.37	0.045	−0.03	0.880	−0.28	0.130	−0.58	0.001	−0.17	0.376	−0.56	0.001
NFC (%CHO)	−0.25	0.187	−0.30	0.109	−0.27	0.146	−0.13	0.489	−0.28	0.140	−0.01	0.965	−0.23	0.224	−0.49	0.006	−0.09	0.619	−0.52	0.003
NSC (%DM)	−0.27	0.154	−0.25	0.185	−0.45	0.012	0.25	0.182	−0.15	0.418	−0.31	0.096	0.10	0.600	0.03	0.855	−0.20	0.282	0.28	0.132

Notes: * DM, dry matter; CP, crude protein; SCP, soluble crude protein; NPN, non-protein nitrogen; NDICP, neutral detergent-insoluble crude protein; ADICP, acid detergent-insoluble crude protein; CHO, total carbohydrate; NDF, neutral detergent fiber; ADF, acid detergent fiber; ADL, acid detergent lignin; Hemi, Hemicellulose (Hemicellulose = NDF − ADF); Cell, Cellulose (Cellulose = ADF − ADL); NFC, non-fiber carbohydrate; NSC, non-structural carbohydrate. ** AI_II_T, Amide I and II peak area; AI, Amide I area; AII, Amide II area; R_AAI_II, Area ratios of amide I and II; AIH, Amide I height; AIIH, Amide II height; R_HAI_II, Height ratios of amide I and II; alpha, α-helix height; beta, β-sheet height; R_Ha_b, Ratio α-helix:β-sheet. *** *r*: correlation coefficient calculated using Spearman method.

**Table 9:** Correlation analyses between protein structure spectral characteristics and estimated energy values of canola seeds.

Items	** AI_II_T		AI		AII		R_AAI_II		AIH		AIIH		R_HAI_II		Alpha		Beta		R_Ha_b	
	*** *r* Value	*p* Value	*R* Value	*p* Value	*r* Value	*p* Value	*R* Value	*p* Value	*r* Value	*p* Value	*r* Value	*p* Value	*r* value	*p* Value	*r* Value	*p* Value	*r* Value	*p* Value	*r* Value	*p* Value
Digestible nutrients % of DM																				
* td NDF	−0.33	0.072	−0.25	0.181	0.11	0.575	−0.32	0.085	−0.12	0.513	0.11	0.547	−0.29	0.120	−0.14	0.453	−0.10	0.616	−0.07	0.724
td NFC	−0.18	0.346	−0.21	0.272	−0.01	0.954	−0.12	0.514	−0.20	0.299	−0.07	0.723	−0.13	0.495	−0.15	0.442	−0.22	0.234	0.26	0.165
td CP	0.08	0.680	0.16	0.394	0.10	0.586	0.00	0.981	0.22	0.241	0.34	0.063	−0.18	0.356	0.22	0.255	0.25	0.183	−0.06	0.771
td FA	0.22	0.242	0.16	0.402	−0.13	0.496	0.29	0.122	0.09	0.624	−0.11	0.561	0.26	0.161	0.05	0.786	0.06	0.748	−0.11	0.578
TDN _1X_	0.21	0.270	0.12	0.525	−0.13	0.498	0.23	0.219	0.07	0.712	−0.05	0.799	0.15	0.442	0.06	0.766	0.01	0.955	−0.01	0.960
TDN p _3X_	0.21	0.270	0.12	0.525	−0.13	0.498	0.23	0.219	0.07	0.712	−0.05	0.799	0.15	0.442	0.06	0.766	0.01	0.955	−0.01	0.960
TDN p _4X_	0.21	0.270	0.12	0.525	−0.13	0.498	0.23	0.219	0.07	0.712	−0.05	0.799	0.15	0.442	0.06	0.766	0.01	0.955	−0.01	0.960
Energy values (Mcal/kg DM)																				
DE _1x_	0.24	0.209	0.16	0.401	−0.10	0.593	0.22	0.241	0.11	0.578	0.00	0.987	0.12	0.523	0.10	0.616	0.05	0.777	−0.02	0.921
DE p _3x_	0.23	0.229	0.14	0.461	−0.13	0.486	0.25	0.193	0.08	0.66	−0.03	0.893	0.13	0.481	0.07	0.708	0.03	0.876	−0.03	0.879
ME p _3x_	0.22	0.244	0.13	0.488	−0.12	0.539	0.22	0.235	0.08	0.679	−0.03	0.892	0.13	0.490	0.07	0.706	0.03	0.898	−0.01	0.948
NEL p _3x_	0.22	0.252	0.15	0.444	−0.13	0.509	0.25	0.182	0.10	0.615	−0.03	0.858	0.15	0.423	0.08	0.685	0.05	0.783	−0.05	0.806
ME _3x_	0.23	0.231	0.15	0.430	−0.12	0.542	0.23	0.219	0.10	0.609	−0.01	0.944	0.13	0.495	0.09	0.645	0.05	0.801	−0.03	0.875
NE m_3x_	0.24	0.209	0.15	0.427	−0.12	0.529	0.24	0.208	0.10	0.614	−0.01	0.945	0.13	0.487	0.09	0.656	0.04	0.818	−0.02	0.904
NE g _3x_	0.22	0.24	0.15	0.440	−0.11	0.547	0.23	0.227	0.10	0.604	−0.01	0.947	0.13	0.504	0.09	0.638	0.05	0.787	−0.04	0.833
DE p _4x_	0.22	0.238	0.15	0.436	−0.11	0.564	0.22	0.242	0.10	0.611	−0.01	0.969	0.12	0.527	0.09	0.647	0.05	0.81	−0.02	0.900
ME p _4x_	0.23	0.223	0.16	0.412	−0.10	0.584	0.22	0.248	0.10	0.591	−0.01	0.977	0.12	0.518	0.09	0.634	0.05	0.792	−0.02	0.905
NEL p _4x_	0.23	0.23	0.15	0.429	−0.11	0.562	0.23	0.231	0.10	0.605	−0.03	0.892	0.14	0.447	0.08	0.666	0.05	0.814	−0.03	0.882

Notes: * td NDF, truly digestible neutral detergent fiber; td NFC, truly digestible non-fiber carbohydrate; td CP, truly digestible crude protein; td FA, truly digestible fatty acid; TDN _1x_, total digestible nutrients at one times maintenance; TDN p _3x_, total digestible nutrients at productive level of intake at three times maintenance; TDN p _4x_, total digestible nutrients at productive level at four times maintenance; DE _1x_, digestible energy at one times maintenance; DE p _3x_, digestible energy at a productive level of intake ( three times maintenance); ME p _3x_, metabolizable energy at production level of intake (3x maintenance); NEL p _3x_, net energy for lactation at productive level (three times maintenance); ME _3x_, metabolizable energy; NE m _3x_, net energy for maintenance; NE g _3x_, net energy for gain. ** AI_II_T, Amide I and II peak area; AI, Amide I area; AII, Amide II area; R_AAI_II, Area ratios of amide I and II; AIH, Amide I height; AIIH, Amide II height; R_HAI_II, Height ratios of amide I and II; alpha, α-helix height; beta, β-sheet height; R_Ha_b, Ratio α-helix:β-sheet. *** *r*: correlation coefficient calculated using Spearman method.

**Table 10:** Correlation analyses between protein structure spectral characteristics and estimated energy values of canola meal.

Items	** AI_II_T		AI		AII		R_AAI_II		AIH		AIIH		R_HAI_II		Alpha		Beta		R_Ha_b	
	*** *r* Value	*p* Value	*R* Value	*p* Value	*r* Value	*p* Value	*R* Value	*p* Value	*r* Value	*p* Value	*r* Value	*p* Value	*r* value	*p* Value	*r* Value	*p* Value	*r* Value	*p* Value	*r* Value	*p* Value
Digestible nutrients % of DM																				
* td NDF	0.05	0.803	0.09	0.623	−0.04	0.848	0.35	0.060	0.09	0.621	−0.24	0.198	0.42	0.019	0.42	0.021	0.02	0.896	0.55	0.002
td NFC	−0.33	0.073	−0.39	0.033	−0.29	0.124	−0.24	0.209	−0.37	0.045	−0.02	0.897	−0.29	0.124	−0.58	0.001	−0.17	0.378	−0.56	0.001
td CP	0.41	0.023	0.42	0.020	0.21	0.267	0.33	0.076	0.43	0.017	0.04	0.842	0.37	0.043	0.61	<0.001	0.31	0.101	0.56	0.001
TDN _1X_	−0.02	0.928	−0.01	0.966	−0.31	0.093	0.48	0.007	0.04	0.848	−0.30	0.102	0.46	0.011	0.21	0.269	0.11	0.581	0.25	0.179
TDN p _3X_	−0.02	0.921	−0.01	0.957	−0.31	0.093	0.48	0.008	0.04	0.849	−0.30	0.101	0.46	0.011	0.21	0.273	0.11	0.579	0.25	0.184
TDN p _4X_	−0.02	0.915	−0.01	0.950	−0.31	0.090	0.48	0.008	0.03	0.858	−0.31	0.099	0.46	0.011	0.20	0.279	0.10	0.588	0.25	0.182
Energy values (Mcal/kg DM)																				
DE _1x_	0.07	0.698	0.09	0.652	−0.24	0.203	0.50	0.005	0.14	0.454	−0.26	0.173	0.49	0.006	0.30	0.106	0.19	0.322	0.29	0.124
DE p _3x_	0.08	0.662	0.09	0.626	−0.24	0.195	0.51	0.004	0.14	0.470	−0.24	0.193	0.48	0.007	0.29	0.124	0.20	0.301	0.28	0.139
ME p _3x_	0.08	0.662	0.09	0.626	−0.24	0.195	0.51	0.004	0.14	0.470	−0.24	0.193	0.48	0.007	0.29	0.124	0.20	0.301	0.28	0.139
NEL p _3x_	0.11	0.552	0.13	0.488	−0.22	0.248	0.51	0.004	0.17	0.359	−0.23	0.228	0.49	0.006	0.32	0.082	0.22	0.248	0.27	0.143
ME _3x_	0.09	0.627	0.09	0.621	−0.25	0.190	0.51	0.004	0.15	0.441	−0.26	0.162	0.49	0.006	0.30	0.104	0.20	0.285	0.29	0.126
NE m_3x_	0.10	0.592	0.11	0.580	−0.24	0.201	0.49	0.006	0.16	0.401	−0.24	0.203	0.48	0.007	0.31	0.094	0.21	0.272	0.29	0.123
NE g _3x_	0.04	0.824	0.05	0.798	−0.28	0.137	0.50	0.005	0.10	0.607	−0.28	0.139	0.48	0.007	0.26	0.159	0.15	0.423	0.28	0.138
DE p _4x_	0.07	0.699	0.09	0.650	−0.26	0.161	0.52	0.004	0.13	0.494	−0.27	0.143	0.49	0.006	0.30	0.103	0.18	0.337	0.30	0.113
ME p _4x_	0.09	0.638	0.10	0.600	−0.24	0.196	0.52	0.003	0.15	0.429	−0.26	0.161	0.51	0.004	0.31	0.094	0.20	0.287	0.28	0.131
NEL p _4x_	0.05	0.810	0.05	0.804	−0.28	0.136	0.50	0.005	0.10	0.609	−0.27	0.144	0.48	0.007	0.26	0.162	0.15	0.423	0.28	0.141

Notes: * td NDF, truly digestible neutral detergent fiber; td NFC, truly digestible non-fiber carbohydrate; td CP, truly digestible crude protein; td FA, truly digestible fatty acid; TDN _1X,_ total digestible nutrients at one times maintenance; TDN p _3X_, total digestible nutrients at productive level of intake at three times maintenance; TDN p _4X_, total digestible nutrients at productive level at four times maintenance; DE _1X_, digestible energy at one times maintenance; DE p _3x_, digestible energy at a productive level of intake (3x maintenance); ME p _3x_, metabolizable energy at production level of intake (3x maintenance); NEL p _3x_, net energy for lactation at productive level (3x maintenance); ME _3x_, metabolizable energy; NE m _3x_, net energy for maintenance; NE g _3x_, net energy for gain. ** AI_II_T, Amide I and II peak area; AI, Amide I area; AII, Amide II area; R_AAI_II, Area ratios of amide I and II; AIH, Amide I height; AIIH, Amide II height; R_HAI_II, Height ratios of amide I and II; alpha, α-helix height; beta, β-sheet height; R_Ha_b, Ratio α-helix:β-sheet. *** *r*: correlation coefficient calculated using Spearman method.

**Table 11:** Multiple regression analyses to find the important protein structural variables for predicting chemical profiles of canola seeds.

Predicted Variables (Y)	Variable (s) Selection (Variables Left in the Model with *p* < 0.05)	Prediction Equation Test Model: Y = a + b1 × x1 + b2 × x2…	*R*^2^ Value	* RSD	*p* Value
** DM%	*** Height left in the model	DM% = 97.44 − 2.01 × Height	0.30	0.80	0.002
Ash (%DM)	No variable met the 0.05 significance level for entry into the model				
EE (%DM)	No variable met the 0.05 significance level for entry into the model				
FA (%DM)	No variable met the 0.05 significance level for entry into the model				
Protein profile					
CP (%DM)	No variable met the 0.05 significance level for entry into the model				
SCP (%DM)	Peak area and AI left in the model	SCP (%DM) = 7.43 + 0.56 × Peak area − 0.97 × AI	1.55	0.62	0.002
SCP (%CP)	Peak area and AI left in the model	SCP (%CP) = 33.81 + 2.69 × Peak area − 4.74 × AI	0.38	5.76	0.001
NPN (%DM)	No variable met the 0.05 significance level for entry into the model				
NPN (%CP)	No variable met the 0.05 significance level for entry into the model				
NPN (%SCP)	Peak area and AI left in the model	NPN (%SCP) = 113.86 − 10.43 × Peak area + 19.24 × AI	0.39	20.86	0.001
NDICP (%DM)	Peak area and AI left in the model	NDICP (%DM) = 2.10 − 0.13 × Peak area +0.24 × AI	0.51	0.20	<0.001
NDICP (%CP)	Peak area and AI left in the model	NDICP (%CP) = 13.50 − 0.55 × Peak area +1.00 × AI	0.51	0.87	<0.001
ADICP (%DM)	No variable met the 0.05 significance level for entry into the model				
ADICP (%CP)	No variable met the 0.05 significance level for entry into the model				
CHO profile					
CHO (%DM)	No variable met the 0.05 significance level for entry into the model				
Sugar (%DM)	No variable met the 0.05 significance level for entry into the model				
Sugar (%NFC)	No variable met the 0.05 significance level for entry into the model				
NDF (%DM)	No variable met the 0.05 significance level for entry into the model				
ADF (%DM)	No variable met the 0.05 significance level for entry into the model				
ADF (%NDF)	No variable met the 0.05 significance level for entry into the model				
ADL (%DM)	Height left in the model	ADL (%DM) = 3.76 + 0.82 × Height	0.14	0.54	0.042
ADL (%NDF)	Height left in the model	ADL (%NDF) = 20.43 + 5.61 × Height	0.21	2.82	0.010
Cell (%DM)	No variable met the 0.05 significance level for entry into the model				
NFC (%DM)	No variable met the 0.05 significance level for entry into the model				
NFC (%CHO)	No variable met the 0.05 significance level for entry into the model				
NSC (%DM)	No variable met the 0.05 significance level for entry into the model				

Notes: * RSD, residual standard deviation. ** DM, dry matter; EE, ether extract; FA, fatty acid; CP, crude protein; SCP, soluble crude protein; NPN, non-protein nitrogen; NDICP, neutral detergent-insoluble crude protein; ADICP, acid detergent-insoluble crude protein; CHO, total carbohydrate; NDF, neutral detergent fiber; ADF, acid detergent fiber; ADL, acid detergent lignin; Cell, cellulose; NFC, non-fiber carbohydrate; NSC, non-structural carbohydrate. *** Protein structural spectral parameters; Height, Height ratios of amide I and II; Peak area, Amide I and II peak area; AI, Amide I area.

**Table 12:** Multiple regression analyses to find the important protein structural variables for predicting chemical profiles of canola meal.

Predicted Variables (Y)	Variable (s) Selection (Variables Left in the Model with *p* < 0.05)	Prediction Equation Test Model: Y = a + b1 × x1 + b2 × x2…	*R*^2^ Value	* RSD	*p* Value
** DM%	*** Alpha left in the model	DM% = 92.49 − 11.13 × Alpha	0.14	0.71	0.039
Ash (%DM)	AIIH and Ratio left in the model	Ash (%DM) = 17.52 − 17.22 × AIIH − 6.89 × Ratio	0.54	0.30	<0.001
Protein profile					
CP (%DM)	Ratio left in the model	CP (%DM) = 30.41 + 11.49 × Ratio	0.32	0.76	0.001
SCP (%DM)	Height and Alpha left in the model	SCP (%DM) = −9.46 + 3.87 × Height +31.26 × Alpha	0.59	0.93	<0.001
SCP (%CP)	Height and Alpha left in the model	SCP (%CP) = −18.24 + 8.19 × Height + 67.45 × Alpha	0.57	2.07	<0.001
NPN (%DM)	AIIH and Height left in the model	NPN (%DM) = −17.41 + 38.07 × AIIH + 8.85 × Height	0.55	0.84	<0.001
NPN (%CP)	AIH and Height left in the model	NPN (%CP) = −21.87 + 38.58 × AIH + 12.52 × Height	0.53	1.88	<0.001
NPN (%SCP)	Peak area, Height and Alpha left in the model	NPN (%SCP) = 39.64 + 1.59 × Peak area + 31.76 × Height − 331.98 × Alpha	0.54	4.04	<0.001
NDICP (%DM)	Alpha left in the model	NDICP (%DM) = 20.99 − 44.95 × Alpha	0.47	1.23	<0.001
NDICP (%CP)	Alpha left in the model	NDICP (%CP) = 51.92 − 113.10 × Alpha	0.48	3.05	<0.001
ADICP (%DM)	No variable met the 0.05 significance level for entry into the model				
ADICP (%CP)	No variable met the 0.05 significance level for entry into the model				
CHO profile					
CHO (%DM)	Height left in the model	CHO (%DM) = 56.83 − 3.08 × Height	0.25	0.62	0.005
Sugar (%DM)	AII, AIIH and Alpha left in the model	Sugar (%DM) = 13.27 − 3.02 × AII + 64.08 × AIIH + 24.02 × Alpha	0.52	0.71	<0.001
Sugar (%NFC)	Ratio left in the model	Sugar (%NFC) = − 11.74 + 43.20 × Ratio	0.24	3.48	0.006
NDF (%DM)	Height and Beta left in the model	NDF (%DM) = 52.87 − 5.79 × Height − 30.05 × Beta	0.29	1.64	0.010
ADF (%DM)	AIIH left in the model	ADF (%DM) = 14.16 + 38.79 × AIIH	0.23	0.86	0.007
ADF (%NDF)	Beta left in the model	ADF (%NDF) = 29.71 + 117.76 × Beta	0.45	2.81	<0.001
ADL (%DM)	Height left in the model	ADL (%DM) = 16.28 − 3.32 × Height	0.19	0.79	0.016
ADL (%NDF)	AII left in the model	ADL (%NDF) = 16.59 + 1.67 × AII	0.14	2.10	0.042
Cell (%DM)	Beta left in the model	Cell (%DM) = 6.93 + 14.56 × Beta	0.31	0.48	0.002
NFC (%DM)	AII and Ratio left in the model	NFC (%DM) = 49.56 − 0.85 × AII − 16.84 × Ratio	0.47	0.91	<0.001
NFC (%CHO)	AIIH and Ratio left in the model	NFC (%CHO) = 95.38 − 72.97 × AIIH − 31.17 × Ratio	0.42	1.75	<0.001
NSC (%DM)	AII, AIIH and Alpha left in the model	NSC (%DM) = 14.27 − 3.02 × AII + 64.08 × AIIH + 24.02 × Alpha	0.52	0.71	<0.001

Notes: * RSD, residual standard deviation. ** DM, dry matter; EE, ether extract; FA, fatty acid; CP, crude protein; SCP, soluble crude protein; NPN, non-protein nitrogen; NDICP, neutral detergent-insoluble crude protein; ADICP, acid detergent-insoluble crude protein; CHO, total carbohydrate; NDF, neutral detergent fiber; ADF, acid detergent fiber; ADL, acid detergent lignin; Cell, cellulose; NFC, non-fiber carbohydrate; NSC, non-structural carbohydrate. *** Protein structural spectral parameters; Height, Height ratios of amide I and II; Peak area, Amide I and II peak area; AII, Amide II area; AIH, Amide I height; AIIH, Amide II height; alpha, α- helix height; beta, β-sheet height; Ratio, Ratio α-helix: β-sheet.

**Table 13:** Multiple regression analyses to find the important protein structural variables for predicting energy values of canola seeds.

Predicted Variables (Y)	Variable (s) Selection (Variables Left in the Model with *p* < 0.05)	Prediction Equation Test Model: Y = a + b1 × x1+ b2 × x2…	*R*^2^ Value	* RSD	*p* Value
Digestible nutrients % of DM					
** td NDF	No variable met the 0.05 significance level for entry into the model				
td NFC	No variable met the 0.05 significance level for entry into the model				
td CP	No variable met the 0.05 significance level for entry into the model				
td FA	No variable met the 0.05 significance level for entry into the model				
TDN _1X_	No variable met the 0.05 significance level for entry into the model				
TDN p _3X_	No variable met the 0.05 significance level for entry into the model				
TDN p _4X_	No variable met the 0.05 significance level for entry into the model				
Energy values (Mcal/kg DM)					
DE _1x_	No variable met the 0.05 significance level for entry into the model				
DE p _3x_	No variable met the 0.05 significance level for entry into the model				
ME p _3x_	No variable met the 0.05 significance level for entry into the model				
NEL p _3x_	No variable met the 0.05 significance level for entry into the model				
ME _3x_	No variable met the 0.05 significance level for entry into the model				
NE m_3x_	No variable met the 0.05 significance level for entry into the model				
NE g _3x_	No variable met the 0.05 significance level for entry into the model				
DE p _4x_	No variable met the 0.05 significance level for entry into the model				
ME p _4x_	No variable met the 0.05 significance level for entry into the model				
NEL p _4x_	No variable met the 0.05 significance level for entry into the model				

Notes: * RSD, residual standard deviation. ** td NDF, truly digestible neutral detergent fiber; td NFC, truly digestible non-fiber carbohydrate; td CP, truly digestible crude protein; td FA, truly digestible fatty acid; TDN _1X_, total digestible nutrients at one times maintenance; TDN p _3X_, total digestible nutrients at productive level of intake at three times maintenance; TDN p _4X_, total digestible nutrients at productive level at four times maintenance; DE _1X,_ digestible energy at one times maintenance; DE p _3x_, digestible energy at a productive level of intake (3x maintenance); ME p 3x, metabolizable energy at production level of intake (3x maintenance); NEL p _3x,_ net energy for lactation at productive level (3x maintenance); ME _3x_, metabolizable energy; NE m _3x_, net energy for maintenance; NE g _3x,_ net energy for gain.

**Table 14:** Multiple regression analyses to find the important protein structural variables for predicting energy values of canola meal

Predicted Variables (Y)	Variable (s) Selection (Variables Left in the Model with *p* < 0.05)	Prediction Equation Test Model: Y = a + b1 × x1+ b2 × x2…	*R*^2^ Value	* RSD	*p* Value
Digestible nutrients % of DM					
** td NDF	*** Ratio left in the model	td NDF = −4.28 + 9.62 × Ratio	0.31	0.65	0.001
td NFC	AII and Ratio left in the model	td NFC = 48.58 − 0.83 × AII − 16.50 × Ratio	0.47	0.89	<0.001
td CP	Ratio left in the model	td CP = 29.40 + 11.60 × Ratio	0.34	0.74	0.001
TDN _1X_	Area left in the model	TDN _1X_ = 55.82 + 2.83 × Area	0.14	0.94	0.040
TDN p _3X_	Area left in the model	TDN p _3X_ = 51.27 + 2.60 × Area	0.14	0.87	0.040
Energy values (Mcal/kg DM)					
DE _1x_	Height left in the model	DE _1x_ = 2.90 + 0.19 × Height	0.20	0.05	0.014
DE p _3x_	Height left in the model	DE p _3x_ = 2.67 + 0.17 × Height	0.19	0.05	0.016
ME p _3x_	Height left in the model	ME p _3x_ = 2.25 + 0.17 × Height	0.19	0.05	0.016
NEL p _3x_	Height left in the model	NEL p _3x_ = 1.39 + 0.12 × Height	0.19	0.03	0.017
ME _3x_	Height left in the model	ME _3x_ = 2.38 + 0.15 × Height	0.19	0.03	0.017
NE m_3x_	Height left in the model	NE m_3x_ = 1.51 + 0.13 × Height	0.18	0.03	0.018
NE g _3x_	Height left in the model	NE g _3x_ = 0.92 + 0.11 × Height	0.19	0.03	0.017
DE p _4x_	Height left in the model	DE p _4x_ = 2.55 + 0.16 × Height	0.19	0.05	0.016
ME p _4x_	Height left in the model	ME p _4x_ = 2.12 + 0.17 × Height	0.19	0.05	0.015
NEL p _4x_	Height left in the model	NEL p _4x_ = 1.31 + 0.11 × Height	0.18	0.03	0.020

Notes: * RSD, residual standard deviation. ** td NDF, truly digestible neutral detergent fiber; td NFC, truly digestible non-fiber carbohydrate; td CP, truly digestible crude protein; TDN _1x_, total digestible nutrients at one times maintenance; TDN p _3x_, total digestible nutrients at productive level of intake at three times maintenance; DE _1x_, digestible energy at one times maintenance; DE p _3x_, digestible energy at a productive level of intake (3x maintenance); ME p _3x_, metabolizable energy at production level of intake (3x maintenance); NEL p _3x_, net energy for lactation at productive level (3x maintenance); ME _3x_, metabolizable energy; NE m _3x_, net energy for maintenance; NE g _3x_, net energy for gain. *** Protein structural spectral parameters; Height, Height ratios of amide I and II; AII, Amide II area; Ratio, Ratio α-helix: β-sheet; Area, Area ratios of amide I and II.
